# Care seeking behavior of people with common mental disorders in São Paulo-Brazil

**DOI:** 10.1186/s13033-020-00369-4

**Published:** 2020-05-24

**Authors:** Gustavo de Brito Venâncio dos Santos, Moisés Goldbaum, Chester Luiz Galvão César, Reinaldo José Gianini

**Affiliations:** 1 Medical and Health Sciences School–PUCSP, Sorocaba, Brazil; 2 Department of Preventive Medicine, University of São Paulo, Medicine School, São Paulo, Brazil; 3 Department of Epidemiology, University of São Paulo, Public Health School, São Paulo, Brazil

**Keywords:** Care seeking, healthcare accessibility, Health equity, Mental health, Health surveys

## Abstract

**Background:**

Mental health in developing countries is a keen area for improvements. Epidemiological research in this field helps to reinforce information, generate hypothesis and guide police makers. This study intends to analyze patterns of care seeking among cases of common mental disorders (CMD) in São Paulo city in 2015.

**Methods:**

The data is from the population-based survey ISA-Capital 2015 and the screening for common mental disorders follows the Self-reporting questionnaire (SRQ-20). The study analyses care seeking according to sociodemographic and health conditions.

**Results:**

The prevalence of CMD was 19.7% (95% CI 18.2–21.4%). There was a higher prevalence of CMD among who sought care in last 30 days (25.4%). Among CMD cases, care seeking presented significant different prevalence ratio (PR) for: women (PR 1.13; 95% CI 1.05–1.2); age 60 years or more (PR 1.13; 95% CI 1.05–1.22) and 30–44 years (PR 1.10; 95% CI 1.01–1.2); brown skin (PR 0.92; 95% CI 0.86–0.97); single or divorced (PR 0.93; 95% CI 0.89–0.99); unemployed (PR 1.06; 95% CI 1.01–1.12); last 15 days referred morbidity (PR 1.3; 95% CI 1.2–1.34); physical disability (PR 1.11; 95% CI 1.06–1.18); and chronic disease (PR 1.15; 95% CI 1.07–1.24).

**Conclusion:**

Identifying vulnerable groups and developing proper public health actions is important to promote equity accessibility. Analysing care seeking behavior among people with CMD is a strong contribution.

## Introduction

Mental illness is present worldwide and generates a huge social and economic impact. A new projection attributed 32.4% of years lived with disability and 13.0% of disability-adjusted life-years to the global burden of mental illness [1]. Developing countries face a high prevalence of these diseases and are not well prepared to handle them [2]. Furthermore, an association between poverty and mental health diseases in these nations has been suggested [3].

Goldberg and Huxley introduced the term Common Mental Disorders (CMD) to describe a set of somatic, depressive and anxiety symptoms that causes important mental impairment [4]. Researches and practitioners have been accessed it through the self-reporting questionnaire (SRQ-20), which is a screening tool for mental disorders recommended by the world health organization to be used in primary care of developing countries [5].

In Brazil, measures of common mental disorders prevalence vary from 17 to 35% [6–11]. The importance of these studies resides on the direction for public health polices and recognition of possible risk factors and vulnerable populations.

Vulnerability patterns and social determinants are parameters to achieve health equity, mainly in mental health [12]. Consequently, the population who needs more assistance for facing social and health inequalities can be prioritized. One way to assure this condition is through healthcare accessibility.

The aim of this research was to investigate care seeking behavior of people with common mental disorders who lived in São Paulo city urban area in the year of 2015.

## Methods

The analysis was made through data from São Paulo city survey ISA-Capital 2015 [13]. It is the third edition of a survey population-based. Participants were residents from the urban area of São Paulo city. Census tracts were clusters and households were primary sample units. Health administrative regions (North, Midwest, Southeast, South and East) were the strata of the county; in each region, based on the 2010 demographic census were elected 30 tracts. Following domains formed the ISA-Capital 2015 sample: 12–19 years (males and females), 20–59 years (males), 20–59 years (females) and ≥ 60 years (males and females). The sample was calculated based on an estimate of 50% prevalence, with 0.10 error, considering a level of 95% of confidence and an effect design of 1.5. In this research 5942 households were sampled; among these, 5469 were visited. To accomplish the objectives of our research a total of 4043 individuals were interviewed, 3732 aged 15 years old or more, with 3619 people being classified according to the self-reporting questionnaire (SRQ20).

The SRQ-20 contains 20 simple “yes or no” questions and is related to the block E on ISA-Capital 2015, classified the common mental disorder (CMD). This questionnaire was validated in Brazil in 1986 by Mari and Williams [14] in a study that compared it to the standardized psychiatric interview. The sensitivity, taking into consideration the cutting-point of 5/6 for men and 7/8 for women, was 83% and specificity 80%, being a good indicator of morbidity. A more recent validation research conducted in 2008 by Gonçalves, Stein and Kapczinski [15] showed a sensitivity of 86.33% and specificity of 89.31%, considering the cutting-point of 7/8 (compared to the structured clinical interview for DSM-IV-TR as gold standard). The discriminatory power was 0.91 and the Cronbach’s alpha 0.86. Moreover, Scafuza et al. in 2009 [16] tested the SRQ-20 validity in the elderly population finding the cutting-point 4/5 with the best sensitivity and specificity for both genders. Therefore, in this research, the cutting points to establish the presence of CMD were: 64-years-old or younger men = 6 or more positive answers; 64-years-old or younger women = 8 or more positive answers [14]; and 65-year-old or older men or women = 5 or more positive answers [16].

The sociodemographic variables were sex, age group, skin color, marital status, education, working status, and income. The health variables were last 15 days referred morbidity, physical disability, mental/intellectual disability, chronic diseases and headache. The variable related with health accessibility were last 30 days care seeking.

First it was performed the care seeking frequency distribution according to CMD cases. The statistical significance of the prevalence ratio in each observation was evaluated through Chi square test corrected by the Satterthwaite’s approximate F test. Second, the analysis verified the association between the sociodemographic or health condition and care seeking in CMD cases. Poisson regression was used for calculating prevalence ratio (PR) and 95% confidence interval.

The analysis took into consideration the stratification, weighted and cluster sampling process. The statistical analysis employed STATA 11 software.

The ethics committee of PUC-SP, Sorocaba Campus, approved this research (CAAE 66296917.0.0000.5373).

## Results

Among the 3619 people sampled, 780 had a positive screening for CMD. Thus, the prevalence of CMD considering sample weights is 19.7% (95% CI = 18.2–21.4%). The description of each question on SRQ20, together with the numbers of positive answers among individuals with CMD, are listed in Table 1.

**Table 1:** Proportion of positive answers for each question of SRQ20 among individuals with CMD

Question	Description	N positive answers	% positive answers
1	Do you often have headaches?	410	53.6
2	Is your appetite poor?	356	45.6
3	Do you sleep badly?	517	66.3
4	Are you easily frightened?	437	56
5	Do your hands shake?	277	35.5
6	Do you feel nervous, tense or worried?	672	86.2
7	Is your digestion poor?	341	43.7
8	Do you have trouble thinking clearly?	412	52.8
9	Do you feel unhappy?	594	76.2
10	Do you cry more than usual?	365	46.8
11	Do you find it difficult to enjoy your daily activities?	399	51.2
12	Do you find it difficult to make decisions?	466	59.7
13	Is your daily work suffering?	269	34.5
14	Are you unable to play a useful part in life?	229	29.4
15	Have you lost interest in things?	382	49
16	Do you feel that you are a worthless person?	195	25
17	Has the thought of ending your life been on your mind?	95	12.2
18	Do you feel tired all the time?	432	55.4
19	Do you have uncomfortable feelings in your stomach?	371	47.6
20	Are you easily tired?	486	62.3

Those who sought care in the last 30 days presented a higher prevalence of CMD (25.4%). Although the CMD prevalence was slightly larger for whose answered yes for availability in the last care seeking, there was no significant difference (Table 2). CMD cases had been successful in 96.8% when sought for care.

**Table 2:** CMD prevalence and its prevalence ratio in accordance with the healthcare accessibility. Sao Paulo, 2015

Variable	CMD prevalence (%)	Prevalence patio	95% confidence interval	p
Care seeking				
Last 30 days	25.4	1.49	1.29–1.71	< 0.001
More than 30 days	17.1	1		
Availability in the last care seeking				
Yes	20	1.29	0.84–2.00	0.234
No	15.4	1		

The analysis of “last 30 days care seeking” among CMD cases showed higher prevalence for sex (females, PR = 1.13; p < 0.001); age (groups of 30–44 years, PR = 1.10; p = 0.027 and 60 years or more, PR = 1.13; p < 0.001); working status (unemployed, PR = 1.06; p = 0.033); last 15 days referred morbidity (yes, PR = 1.3; p = 0.001); physical disability (yes, PR = 1.11; p = 0.001); and chronic disease (yes, PR = 1.15; p = 0.001). The prevalence of “last 30 days care seeking” among CMD cases showed lower for skin color (brown, PR = 0.92; p = 0.007); and marital status (single or divorced, PR = 0.93; p = 0.017). There was no significant difference of “last 30 days care seeking” for education, income, mental/intellectual disability, and headache. (Table 3).

**Table 3:** Analysis of “last 30 days care seeking” among CMD cases according to sociodemographic and health conditions. Sao Paulo, 2015

Variable	Last 30 days care seeking			
	%	Prevalence ratio	p	95% CI
Sex				
Male	30.1	1		
Female	46.4	1.13	< 0.001	1.05–1.2
Age group (years)				
15–29	31.2	1		
30–44	44.7	1.10	0.027	1.01–1.20
45–59	40.6	1.07	0.106	0.98–1.16
60 or more	48.6	1.13	0.001	1.05–1.22
Skin color				
White	46	1		
Black	35.6	0.93	0.143	0.84–1.03
Yellow or indigenous	43.8	0.98	0.892	0.79–1.23
Brown	33.8	0.92	0.007	0.86–0.97
Marital status				
Married	44.4	1		
Single or divorced	35	0.93	0.017	0.89–0.99
Widow	45.8	1	0.824	0.93–1.10
Education				
Undergraduate or more	34.1	1		
9 to 11 years (high school)	38.6	1.03	0.625	0.90–1.18
1 to 8 years (basic)	42.8	1.06	0.331	0.94–1.21
No education	42.5	1.06	0.416	0.98–1.23
Working status				
Employed	38.2	1		
Unemployed	46.8	1.06	0.033	1.01–1.12
Income (minimal wage)				
Until 1	36.5	0.98	0.822	0.84–1.14
+1–2	41.3	1.02	0.806	0.89–1.16
+2–4	41.4	1.02	0.785	0.95–1.16
+4–9	40.2	1	0.892	0.89–1.15
+9	38.9	1		
Last 15 days referred morbidity				
No	28.7	1		
Yes	63.8	1.3	< 0.001	1.20–1.34
Physical disability				
No	32.9	1		
Yes	48.6	1.11	< 0.001	1.06–1.18
Mental/intellectual disability				
No	40.7	1		
Yes	47.2	1.05	0.482	0.91–1.19
Headache				
No	37.9	1		
Yes	43.4	1.04	0.194	0.98–1.1
Chronic disease				
No	24.5	1		
Yes	43.7	1.15	< 0.001	1.07–1.24

## Discussion

The prevalence of CMD in this study was 19.7% (95% CI 18.2–21.4%). A systematic review with meta-analysis made by Steel et al. in 2014 [17] described a global CMD prevalence of 17.6% (95% CI 16.3–18.9%). Thus, the present results is similar with the current literature. However, studies made in rural areas tend to present higher prevalence rates when compared to urban areas, as demonstrated by Silva et al. [18] and Costa and Ludermir [19], who reveal a prevalence of 24.1% and 36% of CMD in rural areas, respectively. Therefore, studies that analyse the health care seeking behaviour of people with CMD in rural areas are important, and their results could be compared with those from our present study in order to verify if the principle of equity is present.

Those who sought care in the last 30 days (28.4%) had a 1.49 prevalence ratio of CMD (95% CI 1.29–1.71). The analysis showed sex, age group, skin color, marital status, working status, last 15 days referred morbidity, physical disability, and chronic disease associated with “last 30 days care seeking” among CMD cases.

The higher prevalence of CMD in those who sought health care in the last 30 days highlighted a specific demand and an opportunity to improve the diagnosis and treatment of these patients. Gonçalves and Kapczinski [20] in 2008 found that 51.1% of who sought care in a primary health unity of South Brazil were diagnosed with some psychiatric disorder. Moreover, these patients had more visits to health units in the last 12 months.

The difference between men and women about care seeking shows a common trend. Women with CMD had a 1.12 more prevalence of care seeking in the last 30 days (95% CI 1.05–1.2) when compared with men with CMD. A study conducted in Canada and published in 2016 [21] demonstrated a gender difference in care seeking, where women reported visiting their primary care provider more frequently than men, both for physical and mental health problems. In Brazil, Pinheiro et al. [22] point out that women refer more morbidity and psychological problems than men and, consequentially, look more for medical services. In other Brazilian study published in 2014 that analyzed the gender difference in care seeking behavior [23] other factors were associated with the non-demand for health services, like opening-time of health care facilities and users working hours.

In United States of America, race and ethnic minorities, like Latinos, blacks and native Americans, have less accessibility to healthcare services when compared to whites [24]. In Brazil, the results of a national health survey made in 2013 [25] found that white people reported less underutilization of healthcare than no-white individuals. In our research, neither black, yellow nor indigenous individuals showed a statistical difference with “last 30 days care seeking” when compared to white individuals. However, brown individuals among CMD cases had a prevalence ratio of 0.93 (95% CI 0.87–0.99), lower than white people. The absence of significant difference according to income and education could be a good new if confirmed by future studies, and could be a positive characteristic of the Brazilian health system implemented since 1991 [24].

Single or divorced people with CMD had a slightly lower seeking behavior when compared to married ones (PR 0.93 95%CI 0.82–0.99); this is consistent with other studies [26, 27]. In the study published by the International Journal of Epidemiology [27], several analyses were made aiming to control possible confounding factors, besides age and sex, which affect the association between health care utilization and marital status. The conclusion was that the association remained after ruling out other possible confounding socio-demographic variables such as educational level, degree of urbanization, country of birth and religious background. However, the authors point towards the need of further investigation with the inclusion of socio-psychological variables to better understand this association. Undoubtedly, the marital status is associated with health care use and seeking behavior. It seems that the existence and maintenance of a relationship have a positive influence on the practices of self-care among individuals.

As expected, people with unfavorable health conditions, like “last 15 days referred morbidity” and chronic diseases, sought more for medical assistance.

Faithfully, there was no difference in the prevalence of CMD considering the availability of healthcare, which may reflect an egalitarian accessibility. However, only one aspect of accessibility was measured—the availability of healthcare services. There are a lot of dimensions when considering accessibility including factors as individual-level, practitioner-level, system-and-process-level, and resource-based or practical-level [28].

## Conclusion

Given the results it is possible to identify vulnerable groups and in this way direct proper attention and further research. People who recently sought care had a higher prevalence of common mental disorders, which can serve as an alert for health practitioners. Furthermore, among people with a positive CMD screening some socioeconomic categories were associated with different care seeking behavior. This is important to understand the specific context of this population and create polices to convey healthcare considering equity.

Regarding the availability of healthcare, one indirect measure of health accessibility, CMD cases did not receive a different treatment when compared with negative CMD group. This is favourable, although should be investigated other aspects of health accessibility in further research.

Despite the sample was representative of population and a trained staff collected the data, self-referred information based the present research, which can be listed as a study limitation. Moreover, the survey study model does not allow establishing a causality. Finally, others factors besides the socio-demographic variables analysed influence the care seeking behaviour. There are individual factors (e.g. recognize the need to seeking care and have knowledge about the facilities availability) and external factors (e.g. transport, distance from healthcare service and communication) that should be considerate.

## Data Availability

The datasets used and/or analyzed during the current study are available from the corresponding author on reasonable request.

## Abbreviations

CMD: Common mental disorders
DSM-IV-TR: Diagnostic and statistical manual of mental disorders fourth edition text revision
SRQ-20: Self-reporting questionnaire (with 20 questions)
PR: Prevalence ratio
PUC-SP: Pontifícia Universidade Católica de São Paulo
95% CI: 95% Confidence interval
